# Annexin/S100A Protein Family Regulation through p14ARF-p53 Activation: A Role in Cell Survival and Predicting Treatment Outcomes in Breast Cancer

**DOI:** 10.1371/journal.pone.0169925

**Published:** 2017-01-09

**Authors:** Diana Hatoum, Daniel Yagoub, Alireza Ahadi, Najah T. Nassif, Eileen M. McGowan

**Affiliations:** 1 School of Life Sciences, Faculty of Science, Faculty of Engineering and IT, University of Technology Sydney, Sydney, New South Wales, Australia; 2 School of Biotechnology and Biomolecular Sciences, University of New South Wales, Sydney, New South Wales, Australia; 3 Faculty of Engineering and IT, University of Technology Sydney, Sydney, New South Wales, Australia; University of South Alabama Mitchell Cancer Institute, UNITED STATES

## Abstract

The annexin family and S100A associated proteins are important regulators of diverse calcium-dependent cellular processes including cell division, growth regulation and apoptosis. Dysfunction of individual annexin and S100A proteins is associated with cancer progression, metastasis and cancer drug resistance. This manuscript describes the novel finding of differential regulation of the annexin and S100A family of proteins by activation of p53 in breast cancer cells. Additionally, the observed differential regulation is found to be beneficial to the survival of breast cancer cells and to influence treatment efficacy. We have used unbiased, quantitative proteomics to determine the proteomic changes occurring post p14ARF-p53 activation in estrogen receptor (ER) breast cancer cells. In this report we identified differential regulation of the annexin/S100A family, through unique peptide recognition at the N-terminal regions, demonstrating p14ARF-p53 is a central orchestrator of the annexin/S100A family of calcium regulators in favor of pro-survival functions in the breast cancer cell. This regulation was found to be cell-type specific. Retrospective human breast cancer studies have demonstrated that tumors with functional wild type p53 (p53wt) respond poorly to some chemotherapy agents compared to tumors with a non-functional p53. Given that modulation of calcium signaling has been demonstrated to change sensitivity of chemotherapeutic agents to apoptotic signals, in principle, we explored the paradigm of how p53 modulation of calcium regulators in ER+ breast cancer patients impacts and influences therapeutic outcomes.

## Introduction

Breast cancer sub-types are defined by their molecular heterogeneity and pathological profiles and therapeutic options, response to treatment, and prognosis are based on the diagnosis and classification of tumors into one of the different sub-types [1]. Resistance to treatment and recurrence of breast cancer eventually occurs in many patients leading to the need for combinational treatments, which are associated with an increase in adverse side effects, decreased quality of life and increased morbidity. Latent recurrence is prevalent, particularly in estrogen receptor α (ERα) breast cancers, and is associated with dormancy after treatment, as reviewed in [2]. Treatment options such as radiotherapy and chemotherapy induce tumor suppressor pathways, such as p53, to facilitate cell cycle arrest and cell death (apoptosis) [3–7]. Novel therapies that mimic p14ARF, a tumor suppressor and an upstream regulator of p53, are now in anti-cancer pre-clinical and clinical trials [8, 9]. Albeit, there is growing evidence to strongly suggest that re-expression of the wild-type p53 (p53wt) protein protects cells from apoptosis [3, 4]. Retrospective human breast cancer studies show tumors with functional p53wt respond more poorly to some chemotherapeutic agents when compared to tumors with non-functional p53 [10–12]. Chemotherapy responses in mouse models with p53wt show induction of growth arrest, and cellular senescence, but not cell death, resulting in minimal tumor regression and early relapse, hence supporting the findings of poorer responses to chemotherapy in the presence of p53wt [4]. Prior studies have suggested that p53 binds to ER as a strategy to prevent apoptosis in ER+ breast cancers [13–15]. Our laboratory has demonstrated that activation of the p53-p21 pathway by p14ARF, in addition to rapid induction of cell cycle arrest, initiated a change in cellular metabolism consistent with a metabolically active senescence-like phenotype most likely to be important in cell survival and recurrence [16]. In our studies, the cancer cells maintained cell viability, and a sub-set of these cells retained the ability to proliferate [16, 17]. Dormant and senescent cells may be more resistant to cancer treatments so the more we understand about the behavior of these cells, the more likely we will be able to understand how cancers develop resistance to current treatments and, importantly, how they recur after treatment.

To gain an understanding of the proteomic fluctuations occurring in breast cancer cells post p14ARF-p53-p21 expression, we employed stable isotope labeling of amino acids in cell culture (SILAC) and tandem mass spectrometry techniques (LC-MS/MS) [18]. This technology allowed the direct comparison of the cellular proteome of breast cancer cells pre- and post activation of the p53 pathway. From the broad based proteomic changes detected, we describe a unique snapshot profile analysis of the differential regulation of the annexin and S100A calcium binding associated protein family members through p14ARF-p53-p21 activation in breast cancer cells. This family of proteins are important regulators of normal cellular function, including cell division, growth regulation and apoptosis [19]. The conserved core calcium/membrane binding unit of these proteins has been described as a means to peripherally tether proteins to membranes, potentially to enable the annexins to organize membranes, thus promoting membrane segregation, vesicle fusion and vesicle trafficking in a calcium dependent manner. Conversely, the unique N-terminus of the individual annexins allows functional diversity [19]. Annexins are consistently deregulated in cancer [20] and particular annexins have been associated with different cancer types and as potential clinical biomarkers [20, 21]. However the literature on dysregulation of annexin protein expression in breast cancer is contentious. Deregulation of individual annexins and S100A proteins have been associated with malignant transformation [22–26], tumor invasion [27–29], metastasis, angiogenesis and drug resistance [20, 30, 31], the effect being dependent on breast cancer sub-type.

This report identifies changes in the annexin and associated S100A family in breast cancer, brought about by p14ARF-p53-p21-activation. Given that individual annexins and S100A proteins have been implicated in cancer initiation and progression, and modulation of calcium signaling has been demonstrated to change sensitivity of chemotherapeutic agents to apoptotic signals [32], we further investigated how the combined overexpression of annexins/S100A proteins, as identified in this study, may contribute to treatment resistance and breast cancer recurrence and metastasis. In principle, we have explored the paradigm of how modulation of calcium regulators through p53 activation may impact on therapeutic options.

## Materials and Methods

### Cell lines and culture

MCF-7 breast cancer epithelial cells (American Type Culture Collection (ATCC) HTB-22) and U2OS osteosarcoma cells (ATCC HTB-96) were stably transfected with p14ARF using the LacSwitch^™^ inducible vector system as previously described [33, 34]. MCF-7p14ARF and U2OSp14ARF cells were selected based on hygromycin B (hB) and Geneticin (G418) resistance respectively. Cells were maintained in hB (200 mg/ml) and G418 (200 mg/ml) to ensure selection of inducible p14ARF. Expression of p14ARF was induced in both cell lines using 5mM tissue culture grade Isopropyl β-D-1-thiogalactopyranoside (IPTG) (Promega) dissolved in phosphate buffered saline (PBS). Cells were routinely cultured in Dulbecco’s Modified Eagle Medium (DMEM, high glucose), with 10% (^v^/_v_) fetal bovine serum (FBS). All cell lines were routinely tested for mycoplasma (Lonza MycoAlert).

### SILAC and LC-MS/MS methodology

The method for SILAC metabolic triple labeling has been described previously [35]. Labeled amino acids, dialyzed fetal bovine serum (FBS), and lysine and arginine-free media (SILAC media) were purchased from Silantes GmbH. Sequencing grade modified porcine trypsin (Promega) was used for all cell passages in labeled medium.

**Fig 1:**
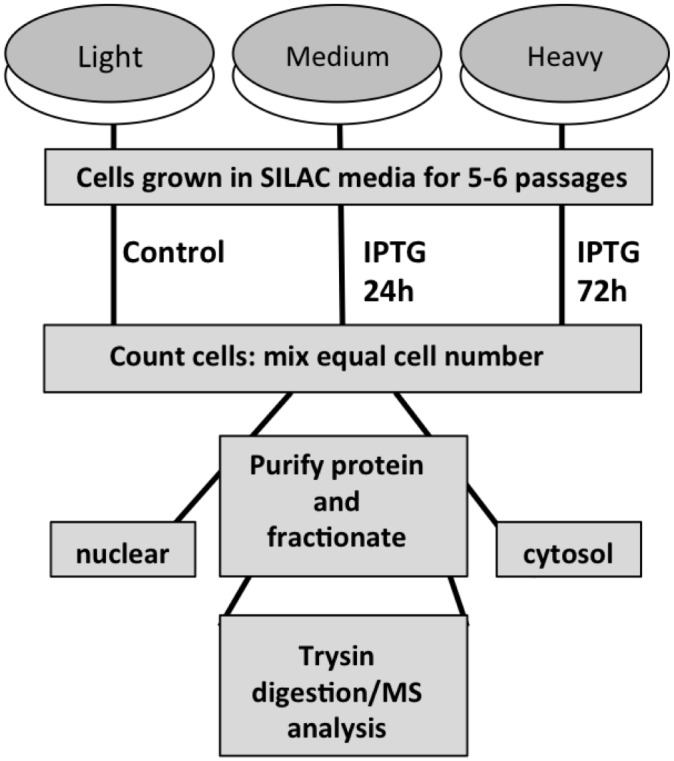
Triple labeling SILAC for proteomic analysis pre- and post p14ARF-p53-p21 activation. MCF-7 cells were split and triple-labeled with three differentially labeled (‘light’, ‘medium’, and ‘heavy’) media formulations as previously described [35]. Proteins were metabolically labeled for a minimum of 6 doublings in lysine- and arginine-free DMEM medium containing 10% (^v^/_v_) dialyzed FBS and supplemented with either: (a) ‘light’, unlabeled lysine and arginine; (b) ‘medium’ lysine-4 (^2^H_4_-lysine) and arginine-6 (^13^C_6_-L-arginine); or (c) ‘heavy’ lysine-8 (^13^C_6_^15^N_2_-L-lysine) and arginine-10 (^13^C_6_^15^N_4_-L-arginine). Cells cultured in ‘medium’ and ‘heavy’ medium were treated with 5mM IPTG for 24h and 72h respectively to induce p14ARF expression. Cells cultured in ‘light’ isotopic medium were treated with PBS. On harvesting, cells were counted and equal numbers of cells were combined at a 1:1:1 ratio. Lysates were fractionated into nuclear and cytoplasmic fractions for better peptide coverage. Cell lysates were separated by electrophoresis and in-gel tryptic digestion was carried out prior to analysis by high-resolution mass spectrometry [35]. Treated cells were harvested, counted and equal numbers of cells were combined from the different SILAC labeled cells in a 1:1:1 ratio and protein extracted. The extracted protein underwent quantitative proteomic analysis by tryptic digestion followed by tandem mass spectrometry (LC MS/MS) [35].

Cells were cultured in SILAC media supplemented with 10% dialyzed FBS and either ‘light’, unlabeled lysine and arginine; ‘medium’ lysine-4 (^2^H_4_-lysine) and arginine-6 (^13^C_6_-L-arginine); or ‘heavy’ lysine-8 (^13^C_6_^15^N_2_-L-lysine) and arginine-10 (^13^C_6_^15^N_4_-L-arginine). Proteins were metabolically labeled in their respective SILAC medium for a minimum of 6 doubling times and stable amino acid incorporation was verified by LC-MS/MS analysis prior to treatments, and demonstrated approximately 96% incorporation of labeled amino acids (data not shown). Cells cultured in ‘medium’ and ‘heavy’ SILAC medium were treated with 5 mM IPTG to induce p14ARF and cells were harvested for protein isolation at 24h and 72h respectively. Cells cultured in ‘light’ SILAC medium were treated with PBS (vehicle) and did not express p14ARF (method outlined in Fig 1).

### Data analysis

To generate a high confidence list of proteins, biological duplicate experiments were performed with the triple labeling strategy, with each biological replicate subsequently being subjected to mass spectrometric analysis twice, producing technical replicates. Mass spectrometric data was processed using MaxQuant software (version 1.0.13.13). MS/MS spectra were searched with the MASCOT search engine against the decoy IPI-human database (forward and reverse sequences) with a peptide and protein false discovery rate of 0.01 as described previously [35]. After identifications at the 1% false discovery rate (FDR) threshold were made, identified proteins were filtered. For inclusion into the filtered dataset, proteins had to be present in both sets of biological replicates, and observed at least twice in technical replicates in duplicate experiments. The STRING database [36], in conjunction with GeneMania [37], was used to analyze the p53/p21/annexin/S100A network associated biological effects. *In silico* pathway-based exploratory multivariate analysis, analyzing associations between the differential annexin regulation seen and treatment outcomes using available patient data from 4142 breast cancer patients with a mean follow up of 69 months, was performed using the Kaplan-Meier Plotter (KMPlot) for breast cancer [38].

### Protein analysis and western blot

Protein isolation and western blot analysis were performed as described previously [17]. Primary antibodies used were p53 (DO-7, Dako, CA, USA), p21 (c-19, Santa Cruz), ANXA A1, ANXA A2 (Becton Dickinson) and β-actin (Abcam), followed by mouse secondary conjugated antibody (Abcam). Protein abundance was quantified by image analysis using the Kodak image station 4000MM.

### Immunofluorescence microscopy

MCF-7p14ARF and U2OSp14ARF cells were cultured on glass coverslips in 6-well plates for 24 h and then treated with 5mM IPTG for 72h. Cells were fixed in freshly thawed 4% paraformaldehyde for 10 min at 37°C, washed with PBS then permeabilized by the addition of cold acetone for 3–5 min at -20°C. Cells were then blocked with 2% (v/v) BSA and 0.1% (v/v) PBS for 1h at RT and labeled with primary antibodies overnight. Primary antibodies were Ki67 (1:400, Abcam, Sapphire) and p14ARF (1:300, Zymed-DKSH). Cells were washed with PBS at RT with gentle rocking for 1 h prior to incubation for 1 h at RT with secondary antibodies. Alexa fluor 568 conjugated anti-mouse IgG (1:500), Alexa fluor 488 conjugated anti-mouse IgG (1:500) and the nuclear stain Hoechst 33342 (trihydrochloride trihydrate 10mg/mL solution in water) (1:1000) were purchased from Invitrogen. Coverslips containing cells were washed and mounted on slides with glycerol based mounting medium. Slides were viewed on a Nikon A1 scanning confocal microscope. Objective specifications were: 60x, oil planApo, 1.40 N/A Perfect Focus System and Differential Interference Contrast (DIC). DAPI (EX 340–380nm), GFP-HQ (EX 420–440nm) and Texas Red (EX 542–580nm) fluorescent filter cubes were used.

### RT-qPCR analysis

RNA was extracted using RNAzol (Molecular Research Center Inc., Cincinnati, OH, USA). RNA (1μg) was reverse transcribed using the High-Capacity cDNA Reverse Transcription Kit (Life Technologies). Quantitative RT-PCR reactions were performed in triplicate in 96-well MicroAmp Fast Optical plates (Applied Biosystems) in a QuantStudio 12K Flex System (Applied Biosystems), using pre-designed and optimized TaqMan gene expression assays (Applied Biosystems). TaqMan Gene Expression Assays used were ANXA1, Hs00167549_m1; ANAX2, Hs01561520_m1; ANXA5, Hs00996187_m1, ANXA6, Hs00XXX_m1 and normalized to GAPDH (Hs02758991_g1) expression. Fold change in expression was calculated by the 2^-ΔΔCt^ method [39].

## Results

### Differential regulation of the expression of the annexin/S100A protein family post p14ARF-p53-p21 activation

**Fig 2:**
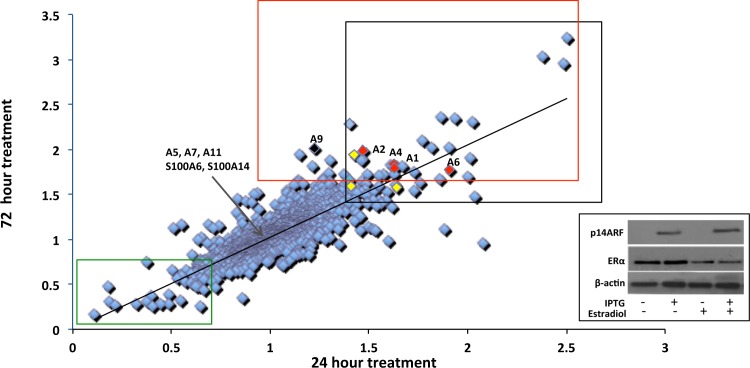
Linear regression analysis of Annexin and S100A protein expression 24h and 72h post p14ARF-p53-p21 activation. MCF-7 cells were treated with IPTG (5mM) to induce the p14ARF-p53 signaling pathway. A filtered set of 1265 proteins was analyzed for two independent biological experiments with technical replicates at 24h and 72h post activation. The correlation coefficient value for the 24h and 72h data for biological duplicate experiments showed a strong correlation (0.79 and 0.72 respectively). The black box highlights proteins significantly over-expressed p<0.05) post p14ARF-p53-p21 activation at 24h and maintained at 72h. The green box highlights proteins significantly downregulated at 24h and maintained at 72h post treatment. The red box highlights proteins significantly upregulated at 72h. The red cubes represent annexin proteins (A1, A2, A4, A6) significantly upregulated at 24h and maintained at 72h; the black cube represents annexin A9 significantly upregulated at 72h (P<0.05). Yellow cubes represent S100A10, S100A11 and S100A13 proteins significantly upregulated at 24h and maintained at 72h. Annexins A5, A7, A11, S100A6 and S100A14 are expressed and not regulated (between ratios 0.8–1.1). Inset: Western blot shows the expression of p14ARF and ER status in MCF-7 cells pre- and post IPTG and β-estradiol treatment at 24 h.

We have previously reported that activation of the p14ARF-p53 pathway in MCF-7 cells leads to cell cycle arrest, without inducing apoptosis, and, additionally, induces a metabolically active senescent-like phenotype [17]. To further explore the underlying mechanisms that lead to cell cycle arrest and metabolic/phenotypic changes we used SILAC LC-MS/MS methodology to determine the proteomic profile of MCF-7 cells post activation of p14ARF-p53 at 24 h and 72 h. Re-expression of p14ARF had no effect on the estrogen response in these cells (Fig 2 inset). Mass spectrometric data processed with MaxQuant software using a stringent filtered dataset, as described in materials and methods, identified 1265 differentially regulated proteins in duplicate experiments. Only proteins identified in biological duplicates in this triple labeling experiment were included to ensure a high confidence list. Linear regression analysis performed on the 1265 proteins demonstrated a strong correlation coefficient value for the 24h and 72h data (0.79 and 0.72 respectively using the two independent datasets) (Fig 2). Most of the proteins did not show a significant difference in expression (Fig 2: 0.7:1.3 ratio). A ratio of <0.7 (downregulated) and >1.3 (upregulated) was considered to be significantly different [40]. Among the top 50 upregulated proteins, annexins A1, A2, A4, A6, S100A10, S100A11 and S100A13 were significantly upregulated at 24h (P<0.05) and 72h (P<0.05). Annexin A9 was upregulated at 72h only (P<0.05). The expression of annexins A5 (an important calcium-dependent regulator of apoptosis), A7, A11, S100A6 and S100A14 remained unchanged at both time points. The SILAC LC-MS/MS analysis of the annexin family and S100A associated proteins is listed in S1 Table.

### Annexin peptide sequences detected by SILAC LC-MS/MS map to unique N-terminal regions of annexin proteins

**Fig 3:**
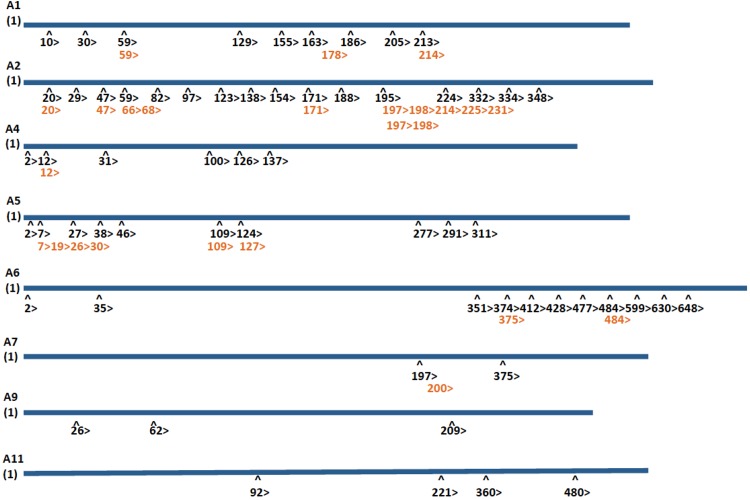
Alignment of annexin peptides identified by MaxQuant with the annexin protein sequences. Unique annexin peptide sequences identified by SILAC-based mass spectrometric analysis were aligned to the N-terminal region of annexins A1, A2, A4, A5, A6, A7, A9, and A11. The number 1 represents the start “M” (methionine) codon, or first amino acid of the protein. Each arrow identifies a unique peptide identified by SILAC LC/MS/MS. The numbers in black indicate the start of the peptide and the orange numbers indicate the overlap of different peptide sequences.

There is a high degree of sequence similarity among the annexin protein family members. The annexin proteins retain a conserved core structural region, containing a Ca^2+^ binding site, and responsible for the Ca^2+^-dependent binding of the proteins to phospholipids, however, individual annexin family members possess unique N-terminal domains, a feature underscoring the functional diversity of individual annexins [19]. Annexin peptides identified by SILAC LC-MS/MS analysis (Table 1) were mapped to the annexin protein sequences using the Clustal Omega and sequence coverage of the annexins ranged from approximately 12–50% (Table 1). The alignment of the annexin sequences listed in the SILAC LC-MS/MS data demonstrated the positioning of peptides to the N-terminal region, which contains amino acid sequences unique to individual annexin family members (Fig 3). No overlap of the annexin peptides, as identified by SILAC/MS/MS, was observed confirming the unique identification and differential regulation of individual annexin family members through the activation of p14ARF-p53-p21.

**Table 1:** SILAC data showing differential expression of the Annexins and associated S100A proteins at 24 and 72 hours post p14ARF induction.

ID	Protein	24h Ratio M/L	72h Ratio H/L	No. of Peptides	Sequence Coverage (%)
IPI00218918	**A1**	1.6	1.8	14	49.7
IPI00418169	**A2**	1.5	2.0	35	71.7
IPI00793199	**A4**	1.6	1.8	7	24.9
IPI00329801	**A5**	1.1	1.1	17	44.4
IPI00221226	**A6**	1.9	1.8	19	13.1
IPI00002460	**A7**	1.1	1.2	5	12.3
IPI00008714	**A9**	1.2	2.0	6	20.3
IPI00414320	**A11**	0.9	1.2	5	10.6
IPI00395627	**S100A6**	0.8	0.8	6	33.8
IPI00183695	**S100A10**	1.4	1.9	3	32.0
IPI00013895	**S100A11**	1.4	1.6	9	63.8
IPI00016179	**S100A13**	1.6	1.6	12	28.6
IPI00010214	**S100A14**	0.99	1	4	46.2

A1–A11 = Annexins A1–A11; S100A = S100A calcium binding protein. Grey highlights indicate no significant regulation at 24h post p14ARF induction (significance threshold ≥ 1.4).

### Differential regulation of annexin expression in MCF-7 cells post p14ARF-p53 activation occurs at the transcriptional level

**Fig 4:**
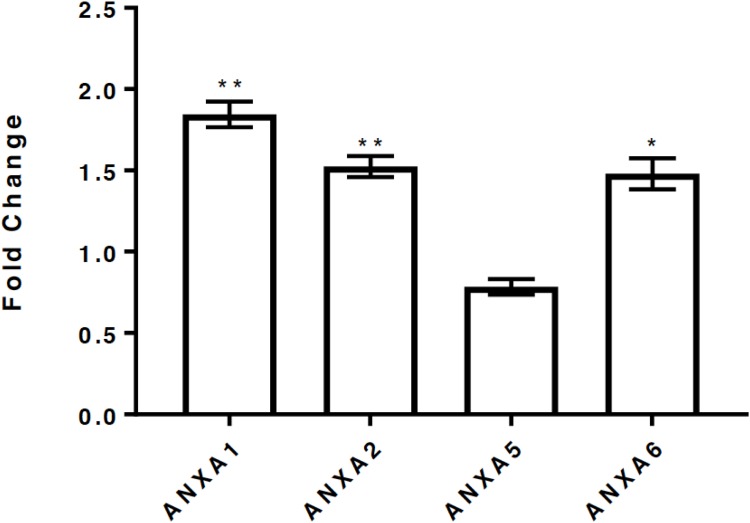
Differential regulation of Annexin A1, A2, A5 and A6 expression at the transcriptional level in MCF-7 breast cancer cells. p14ARF expression was induced by the addition of 5mM IPTG for 15h. Quantitation of ANXA1, ANXA2, ANXA5 and ANXA6 expression was analyzed at 15h post p14ARF induction using the Taqman fast master mix and pre-optimized primer and probe sets. Data were normalized to levels of the reference gene glyceraldehyde-3-phosphate dehydrogenase (GAPDH). Data have been expressed as fold change in expression post p14ARF induction by IPTG at the 15h time point relative to control (2^−ΔΔCt^). Experiments were performed in duplicate in which each set of experiments contained technical triplicates. Statistical differences between groups were determined using a two tailed, paired t-test. *p < 0.02, **p < 0.003 respectively.

To determine if the changes in the annexin protein expression observed were reflected at the transcription level, RT-qPCR was performed at 15h post-p14ARF-p53 induction using specific annexin Taqman probes. The results showed significant upregulation of ANXA1, ANXA2 and ANXA6 (P<0.01) mRNA expression, whereas ANXA5 expression remained unchanged (Fig 4). This is consistent with the SILAC protein quantitation data, which showed upregulation of A1, A2 and A6 proteins with no change in the A5 protein level (Table 1).

### Functional significance of alterations in the p53-p21-annexin network signaling

**Fig 5:**
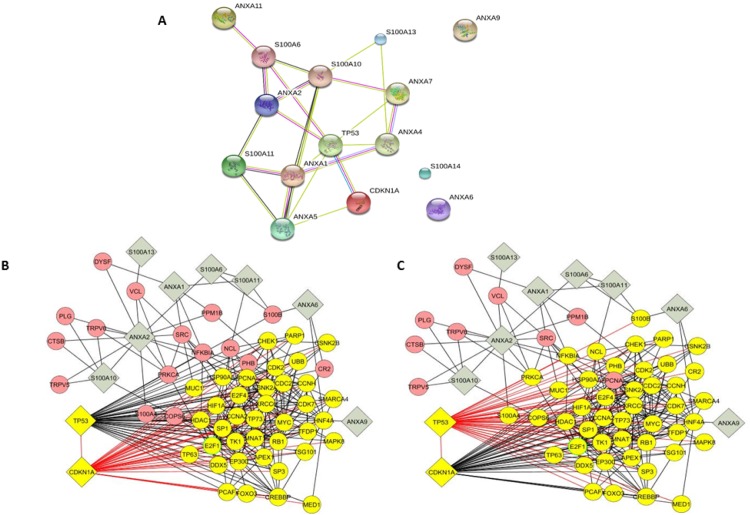
Schematic representation of the p53/p21/annexin/S100A interactome. TP53 and CDNK1A, and their association with ANXA A1, A2, A4, A5, A6, A7, A9, A11 and S100A -6, -10, -11, -13 and -14 were analyzed using the STRING database (v10.0) and the Cytoscape Mimi PLUGIN. (A) Each node represents all the proteins produced by a single protein-coding gene locus, whereby a small node signifies a protein of unknown 3D structure and a large node denotes a protein where some 3D structure is known or predicted. Colored nodes indicate query proteins and first shell interactors. Colored edges (lines) represent interactions between proteins: light blue—known interactions from curated databases; purple—experimentally determined interactions; green—predicted interactions between gene neighborhood; red—gene fusions; dark blue—gene co-occurrence; light green–represents text mining; black—co-expression; grey—protein homology. (B) and (C) Highlighted in yellow are the direct common interactions between CDNK1A and TP53. The annexins and S100A interactions are highlighted in grey. Linkages were analyzed using two methods. (B) TP53 interactions: black lines, CDKN1A red lines. (C) TP53 red lines and CDKN1A black lines (summarized in S2 Table).

As we have strong evidence implicating p53 in the control of annexin and S100A protein expression, we sought to determine how changes in the expression of this family of proteins could influence cell physiology. Using the STRING (v10) database we first searched for strength of the interactions between TP53 (p53) and CDKN1A (p21) and the 13 annexin and S100A proteins. The predicted interactome scores for the protein-protein interactions are shown in the score ladder in Table 2. Unsurprisingly, there is a strong relationship between TP53 and CDKN1A as shown in the score ladder (Table 2), with a very high score of 0.999 (the highest predictive score being 1.0). ANXA1, ANXA2 and ANXA4 directly and strongly interact with TP53 with scores of 0.871, 0.946 and 0.867 respectively. Although ANXA5 is the annexin protein most commonly associated with p53 function, the predicted interacome scores for both TP53 and CDKN1A revealed a lower score of 0.744. ANXA6 and ANXA9 did not reveal direct interaction with TP53 and CDKN1A (Fig 5A). Therefore, the prediction from the STRING analysis demonstrated that not all the ANXAs/S100A family interacted directly with TP53 or CDKN1A.

**Table 2:** p53/p21/annexin/S100A interactome scores for protein-protein interactions from the STRING database.

Protein 1	Protein 2	Accession for protein 1	Accession for protein 2	^#^Score
TP53	CDKN1A	ENSP00000269305	ENSP00000244741	0.999
S100A10	ANXA2	ENSP00000357799	ENSP00000346032	0.999
ANXA2	S100A10	ENSP00000346032	ENSP00000357799	0.999
S100A11	ANXA1	ENSP00000271638	ENSP00000257497	0.983
TP53	ANXA2	ENSP00000269305	ENSP00000346032	0.946
TP53	S100A6	ENSP00000269305	ENSP00000357708	0.930
TP53	ANXA1	ENSP00000269305	ENSP00000257497	0.871
TP53	ANXA4	ENSP00000269305	ENSP00000377833	0.867
S100A6	ANXA11	ENSP00000357708	ENSP00000265447	0.842
S100A6	ANXA2	ENSP00000357708	ENSP00000346032	0.776
TP53	ANXA5	ENSP00000269305	ENSP00000296511	0.744

^#^Note: The highest predictive score is 1.0.

Further, we used the Cytoscape platform to determine the TP53 and CDKN1A and annexin interactive functions. These are predicted functions drawn from known interactions from curated databases or have been experimentally determined. Functional analysis showed TP53 and CDKN1A share many common interactions as distinctly shown in Fig 5B and 5C, and S2 Table. Again using this program we show a close association between all the ANXAs/S100A and TP53 and CDKN1A with only 2–3 degrees of freedom (Fig 5B and 5C). Specifically, these are illustrated by CR2 and HNF4A, which directly interact with TP53 and CDKN1A and directly interact with ANXA6, however ANXA6 does not directly interact with either TP53 or CDKN1A (Fig 5B and 5C and S2 Table). Similarly, ANXA9 directly interacts with HNF4A by not CDKN1A or TP53. The full gene description, function and processes of the p53-p21-annexin/S100A interactome are presented in S2 Table.

### Predicted changes in cellular dynamics associated with annexin/S100A expression post p53 activation

The annexins are calcium-dependent phospholipid binding proteins and Metscape analysis of the annexin/S100A families identified in this report showed similar and compensatory functions of the family members as outlined in Table 3. The annexin family members and the S100A binding proteins have shared protein domains and the GeneMANIA program was used to predict potential degeneracy, or substitution through alternative physical interaction, co-localization and genetic interactions (Table 4). Changes in annexin binding or interacting partners lead to changes or alterations in cellular function, dependent upon the annexin in question. An example from this report is that of annexin A2, predicted to physically interact with both S100A10 and S100A6. However, p53 activation leads to a preferential increase in A2 and S100A10 levels, therefore, in the absence of increases in any of the other A2 binding proteins, A2 would preferentially bind to S100A10. Consequently, the formation of increased levels of the A2/S100A10 complex will promote the cellular functions mediated by this complex, which is to bind to cytoskeletal components associated with intracellular fusion [41]. Annexin A4, also upregulated through p53 activation, is also associated with membrane fusion [42]. Whether A4 and A2/S100A10 are complementary or compensatory mechanisms is yet to be shown.

**Table 3:** Annexin protein function(s) analyzed using the MetScape Plugin in Cytoscape.

Protein	Functions
**A1**	Calcium-dependent phospholipid binding, phospholipid binding, lipid binding, extracellular organelle, extracellular vesicle exosome, extracellular membrane-bounded organelle, lipase inhibitor activity, vesicle fusion.
**A2**	Calcium-dependent phospholipid binding, phospholipid binding, lipid binding, extracellular organelle, extracellular vesicle exosome, extracellular membrane-bounded organelle, vesicle fusion.
**A4**	Calcium-dependent phospholipid binding, phospholipid binding, lipid binding, lipase inhibitor activity, calcium ion binding.
**A5**	Calcium-dependent phospholipid binding, phospholipid binding, lipid binding, extracellular organelle, extracellular vesicle exosome, extracellular membrane-bounded organelle, lipase inhibitor activity.
**A6**	Calcium-dependent phospholipid binding, phospholipid binding, lipid binding, extracellular organelle, extracellular vesicle exosome, extracellular membrane-bounded organelle.
**A7**	Calcium dependent protein binding, lipid binding.
**A9**	Phospholipid binding.
**A11**	Calcium dependent protein binding.
**S100A6**	Calcium dependent protein binding, calcium ion binding, regulation of fibroblast proliferation, ruffle.
**S100A10**	No independent function listed
**S100A11**	Ruffle
**S100A13**	Calcium ion binding

Grey shading denotes no change in annexin and S100A protein expression.

**Table 4:** Cytoscape (GeneMANIA) analysis of Annexin and associated S100A partner overexpression: defining the consequent physical interactions, co-localization and genetic interactions post p14ARF induction.

Protein	Physical interacter(s)	Co-localization	Genetic interactions
**A1**	S100A11	S100A11, A11	A2
**A2**	S100A10, S100A6	NO	A1
**A4**	NO	A9	A5, A11
**A5**	NO	S100A10	A4
**A6**	NO	NO	NO
**A7**	S100A10	NO	NO
**A9**	NO	A4	NO
**A11**	S100A6	A1, S100A11	A4, A11
**S100A10**	A2 and A7	A10	A2
**S100A11**	A1	A1, A11	NO
**S100A13**	NO	NO	NO

All annexin and associated S100A proteins are co-expressed. Annexins have shared protein domains and the S100A proteins have shared protein domains. Physical interactions, co-localization and genetic interaction analysis was completed using Cytoscape with the GenMANIA plug in. Grey shading highlights proteins that are not regulated by ARF-p53.

### Regulation of annexin expression is cell type specific

**Fig 6:**
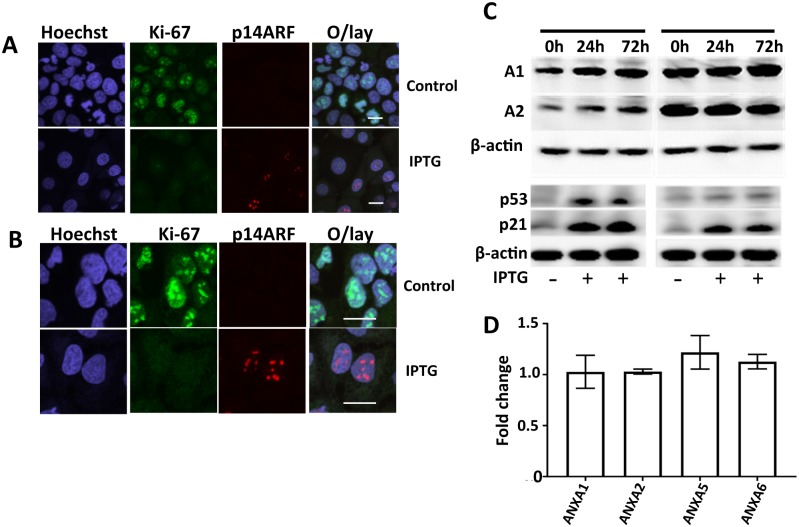
Differential regulation of Annexin A1, A2, A5 by p14ARF-p53 occurs in MCF-7 but not in U2OS osteosarcoma cells. p14ARF expression was induced in MCF-7p14ARF and U2OSp14ARF cells by the addition of 5mM IPTG at? 15h 24h and 72h, PBS was added to control cells. Expression and localization of p14ARF expression in (A) U2OS cells, (B) MCF-7 pre- and post IPTG induction using immunofluorescence microscopy. (C) Top panel shows a representative western blot analysis of annexin A1 and A2 protein expression in MCF-7 and U2OS cells at 24h and 72h time points after p14ARF induction. β-actin was used as a loading control. The MCF-7p14ARF and U2OSp14ARF protein samples shown are from the same western blot. Bottom panel, P21, a p53 transcriptional target, was used to confirm p53 activation in the cell lysates. β-actin was used as a loading control. (D) Gene expression of p14ARF was induced by the addition of 5mM IPTG for 15h in U2OSp14ARF cells. Vehicle control contained PBS in place of IPTG. Transcriptional regulation of ANXA1, ANXA2, ANXA5 and ANXA6 was analyzed at 15h post p14ARF induction using RT-qPCR. Data were normalized to the reference gene glyceraldehyde-3-phosphate dehydrogenase (GAPDH). Data have been expressed as fold change in expression post p14ARF induction relative to the control (2^−ΔΔCt^). Experiments were performed in duplicate in which each set of experiments contained technical triplicates. Statistical differences between groups were determined using a two tailed, paired t-test.

To determine if annexin expression was regulated by p53 in other cell types, we examined the effects of p53 activation in two distinct and functionally different cell lines, U2OS, an osteosarcoma cell line and MCF-7 breast cancer cells. Immunofluorescence microscopy showed P14ARF expression in the nucleolus in both cell lines post IPTG treatment (Fig 6A and 6B). p14ARF expression correlates with downregulation of the proliferation marker Ki-67 in the same cells (Fig 6A and 6B). We compared annexin A1 and A2 protein expression, both of which are important regulators of normal breast cell physiology [41, 43, 44], breast cancer progression and are associated with more aggressive and invasive cancer phenotypes [29]. Using specific annexin (A1 and A2) antibodies, we detected p14ARF-p53 induced upregulation of A1 and A2 proteins in MCF-7 cells (Fig 6C) but no significant change in these protein levels was observed in the U2OS p14ARF cells. Interestingly, higher basal levels of A1 and A2 were detected in U2OS cells compared to MCF-7 cells (Fig 6C). Activation of p14ARF-p53-p21 was confirmed by western blot analysis of p53 and p21 in the same experiments (Fig 6C). This lack of annexin regulation by p53 in U2OS cells was also confirmed at the transcriptional level by RT-qPCR analysis (Fig 6D). Differential annexin regulation was unique to MCF-7 cells and not a common feature of other cell types. This adds further evidence that regulation of annexin expression by p14ARF-p53 is strongly associated with specific annexin-mediated cell functions. Interestingly, one common feature of both cell lines (U2OS and MCF-7) was that calcium dependent apoptosis through annexin A5 was not regulated by p53 at either the transcriptional or translational level.

### Association between p53 induced annexin-S100A overexpression and treatment outcomes in breast cancer: Analysis by individual protein expression status

**Fig 7:**
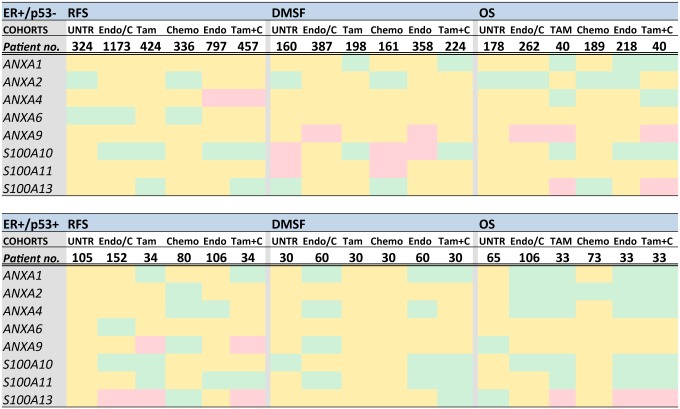
Effect of p53 induced upregulation of individual annexins on patient prognosis. Kaplan-Meier analysis [38] was used to predict breast cancer patient survival (RFS, DMSF and OS) post p53-upregulation of individual annexins: ANXA1, ANXA2, ANXA4, ANXA6, ANXA9 and S100A10, S100A11 and S100A13. A comparison was made between ER+ patients (A), and a sub-set of patients with ER+/p53wt (B). A hazard ratio of 95% confidence intervals and the log-rank P-value (P<0.05) was determined for differences in survival for each treatment option outcome and the results are represented as a heat chart: green = positive prognosis; pink = negative prognosis and yellow neutral (the median was used as a cutoff). The number of patients in each treatment sample is shown. Abbreviations: RFS = Relapse free survival; DMFS = Distant metastasis free survival; OS = Overall survival; UNTR = untreated; Endo = endocrine; Tam = Tamoxifen, C and chemo = chemotherapy.

Dysregulation of the expression of individual annexins has been associated with breast cancer development and poor prognosis, and modulation of calcium signaling by p53 has been associated with lack of sensitivity to chemotherapy and radiotherapy [20, 22, 27, 45–49]. Pre-clinical validation of prognostic gene candidates in a large independent cohort is a prerequisite for the development of robust biomarkers. To evaluate the potential prognostic value of elevated annexin/S100A protein levels, as identified in this study, we performed a meta-analysis using a publicly available breast cancer patient mRNA expression database (accessed from the website *Kmplot*.*com*) [38]. The available breast cancer patient data from the Kaplan Meier website, which consists of 4142 patients with a mean follow up of 69 months, was categorized in treatment sub-groups as indicated in Fig 7A and the number of patients in each cohort is also provided. Cohorts were also compared to a filtered sub-set of patients with ER+/p53wt status (Fig 7B), to determine if patients harboring p53wt had a better or poorer prognosis. We aimed to assess how p53 induced upregulation of annexin/S100A would affect the following clinical outcomes: Relapse Free Survival (RFS), Distant Metastasis Free Survival (DMFS), and Overall Survival (OS), both in untreated and treated ER+ (luminal A) breast cancer patients. The first set of Kaplan-Meier plot analyses were conducted to determine how elevated expression of individual annexins and S100A family members (i.e. ANXA1 ANXA2 ANXA4 ANXA6 ANXA9 S100A10 S100A11 S100A13) would influence response of ER+ patient cohorts to conventional treatments (Fig 7A). Associations between individual annexin/S100A expression data and predicted clinical outcome were categorized according to patient treatment regimes of (A) combined endocrine therapy and chemotherapy (Endo/C), (B) tamoxifen therapy (Tam), (C) chemotherapy alone (Chemo), (D) endocrine therapy alone (Endo), (E) combined tamoxifen and chemotherapy (Tam+C) and (F) untreated (UNTR). All finding and associations are summarized in Fig 7. For simplicity, the predicted survival data has been portrayed as a heat chart whereby the green bars represent a positive prognosis; a negative prognosis by the pink bars and a neutral or non-significant outcome represented the yellow bars (Fig 7).

Using the Kaplan-Meier plot for breast cancer treatment outcome, there were distinct differences in how patients responded to specific therapies dependent on their annexin gene expression profile (Fig 7). In general a more positive DMSF and overall outcome was predicted in the sub-set of patients where tumors were documented as expressing ER+/p53wt. With reference to Fig 7A, only expression of annexin A9, and S100A13 predicted poor overall survival when endocrine therapies were used for treatment. In the sub-set of patients that expressed ER+/p53wt, only S100A13 was shown to be a predictor of poor overall survival (Fig 7B).

### Association of p53 induced annexin-S100A overexpression and breast cancer treatment outcome: Analysis by combined annexin expression status

**Fig 8:**
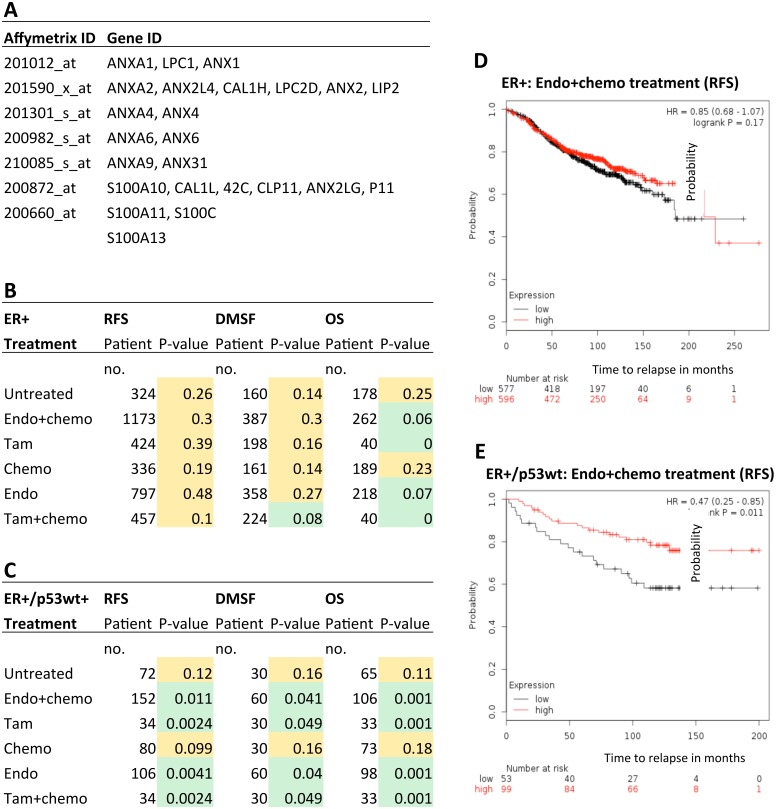
Effect of p53 induced upregulation of all annexin and S100A genes (annexin expression profile) on ER+ patient prognosis. Kaplan-Meier analysis [38] was used to predict breast cancer patient survival (RFS, DMSF and OS) post p53-upregulation of all combined annexins/S100A genes (Fig 8A). A comparison was made between ER+ patients (B), and a sub-set of patients with ER+/p53wt (C). A hazard ratio with 95% confidence intervals and the log-rank P-value (P<0.05) was determined for differences in survival for each treatment option outcome and the results are represented as a heat chart: green = positive prognosis; pink = negative prognosis and yellow neutral (the median was used as a cutoff). The number of patients in each treatment sample is shown. (D) and (E) show comparative representative Kaplan-Meier plots of relapse free survival (RFS) for ER+ (D) and ER+/p53wt+ (E) patients treated with Endo + chemo therapies. The x-axis shows the number survival months from diagnosis. The red line represents patients with high annexin/S100A expression, and the black line represents patients with low annexin/S100A expression.

To determine whether cluster analysis of the differentially regulated annexins and S100A genes (Fig 8A) would confer any concessionary changes on patient prognostic outcome, we used an unbiased combinational approach, which assessed treatment outcome based on the overall (combined) annexin and S100A overexpression profile in ER+ breast cancer patients (Fig 8B). In comparison to the analysis of individual annexins, the first observation was that, overall, patient prognosis was significantly improved when all upregulated gene expression changes (annexin + S100A) were taken into account, and this was independent of treatment regime (compare Figs 7 and 8B). The most striking difference was when the ER+/p53wt+ sub-group was analyzed with the same parameters. Significantly improved outcomes (RFS, DMSF and OS) were observed with tamoxifen treatment alone, or when tamoxifen was included in the treatment regime (compare Fig 8B, 8C, 8D and 8E). Representative Kaplan-Meier plots comparing the difference in ER+ patient outcome (Fig 8D) with the ER+/p53wt+ (Fig 8E) patient subset when patients are treated with endo+chemo whereby a significant improvement in time to relapse is observed in the presence of p53.

## Discussion

Approximately two-thirds of all breast cancers harbor the wild type p53 protein. Contrary to a long-held belief that favorable chemotherapy outcome is dependent upon p53-mediated apoptosis [50], some reports suggest a less favorable outcome for p53wt breast cancers [4, 15]. Our previous studies have demonstrated that p53 induces a viable, metabolically active senescence-like cellular phenotype which supports the paradigm that p53 may be protective against apoptosis in breast cancer cells [16, 17]. This has been partly explained by the concept that p53 interacts with ER to protect cells against apoptosis, yet how p53 activity hinders chemotherapy response is not clear. In order to accurately predict clinical response, we need to understand the cellular changes occurring in response to activation of the p53 pathway. Regulating calcium signaling is essential for mammary gland function and deregulation of calcium homeostasis is associated with cancer pathophysiology. It has been difficult to determine how these calcium-dependent multi-faceted annexin proteins are regulated due to the sequence similarity of the annexin family of proteins and their compensatory functions within the cell. However, using SILAC LC-MS/MS methodology, we could identify unique peptides within the N-terminal region of the individual annexin proteins and show how p53 regulates the expression of members of this protein family. Our bioinformatic analysis of p53-induced upregulation of protein expression showed a strong association between ANXAs/S100A and either TP53 (p53) or CDKN1A (p21). This aligns with previous findings showing that p53 transcriptional regulation of p21 is a link to its pro-survival function and is opposed to the A5 induced cell death, reviewed in Clarke et al, 2015 [51]. These findings support a renewed study of p53 as a central regulator of normal cellular function and pathophysiology. This report is the first to demonstrate p14ARF-p53 as a key central orchestrator of the annexin/S100A family of calcium regulators in favor of pro-survival functions in the breast cancer cell, in contrast to the activation of the canonical annexin A5 pro-apoptotic response usually associated with this tumor suppressor function. In the two cell lines studied, the annexin A5 pro-apoptotic pathway was not activated by p14ARF-p53.

The annexins A1, A2, A4, A6 and A9, and annexin binding proteins S100A10, S100A11 and S100A13 were in the top 50 proteins upregulated by p14ARF/p53, as evidenced by SILAC-based analysis. Although the function(s) of each annexin is not clearly defined, annexin-Ca^2+^ regulation is unquestionably important in a wide range of both intra- and extracellular functions that require interaction with the acidic phospholipids of the intracellular compartment of all membranes and Ca^2+^ signaling [19].

### Annexins in normal physiology and breast cancer

The annexin A2/S100A10 complex, the abundance of which is increased by p53 activation, plays a role in membrane organization, membrane trafficking, in promoting ion conductance across membranes [19], and in calcium redistribution from bone to breast [52–54]. Annexin A4 has recently been shown to be involved in plasma membrane remodeling, through regulation of the actin cytoskeleton, and in cellular cholesterol homeostasis [55]. The role of annexin A6 as a membrane organizer is further supported by a recent study [56]. These observations are consistent with the changes we have observed in the architectural reorganization of the cytoskeleton of MCF-7 cells post p14ARF/p53/p21 activation [17], suggesting annexin regulation via this pathway may be a normal cellular process in breast physiology.

Aberrant calcium signaling is often linked to each of the hallmarks of cancer cells [57]. In this report we highlight how differential changes in annexin and S100A expression may impact on signaling pathways and potentially lead to the activation or inhibition of downstream and/or compensatory cellular mechanisms, dependent upon the direction of expression change. Annexin and S100A deregulation has been associated with tumor invasion and metastasis, angiogenesis and drug resistance [20, 30, 31]. Loss of annexin A1 has been associated with malignant transformation in ER+ breast cancer [22], and, conversely, recent reports associate high annexin A1 expression with cellular invasion in ER- [27]. Increases in annexin A2 and S100A11 are associated with cell viability and increased invasiveness through their ability to maintain plasma membrane integrity [58] and promote epithelial-mesenchymal transition [29]. Dysregulation of individual annexin expression is associated with cancer development and treatment outcomes and it has been suggested that considering the expression of individual annexins may provide useful diagnostic and prognostic biomarkers [20]. Furthermore, modulation of calcium signaling has been demonstrated to change sensitivity of chemotherapeutic agents to apoptotic signals. This led to our further investigation of the impact of the differential regulation of annexin expression by p53 on patient treatment outcomes.

### The ER-p53-annexin expression profile and treatment outcomes

To address how increases in the expression of individual annexins (A1, A2, A4, A6 and A9) and S100A binding partners (S100A10, S100A11 and S100A13), and combinations of thereof, could influence treatment outcomes, we performed a meta-analysis (biomarker assessment) based on 4142 breast cancer samples using the Kaplan-Meier plot database for breast cancer (available online) [38]. This is the first biomarker analysis directly comparing patient treatment outcomes using expression data of each individual annexin and then combining the expression date of all annexins and S100A binding proteins (i.e. an annexin expression profile) in a specific sub-set of breast cancer patients (ER^+^p53^+^) within a larger cohort. Overall, ER^+^ patient prognosis was more favorable when p53wt was present and was associated with increased RFS, DMSF and OS. The exception to this was upregulation of annexin A9 and S100A13, which were associated with poor RFS and RFS/OS respectively, and, interestingly, this was only in patients who had undergone endocrine treatments. The most favorable prognosis and survival odds were observed when all the upregulated annexins and S100A proteins were taken into account together as an expression profile or signature, and a comparison was made between ER^+^p53^-^ patient tumors and ER^+^p53wt^+^ tumors. In general, all tumors responded more positively when p53wt was expressed independent of treatment regime. The most striking observation was that of ER^+^p53^+^ tumors with the expression profile of upregulated annexin A1, A2, A4, A6, A9 and S100A, A11 and A13, which showed great benefit from tamoxifen intervention alone, and, it was further shown, that additional treatment with chemotherapy would have no added beneficial effect. *In conclusion*, this study ascribes to p53wt the functions of a key organizer of calcium metabolism in breast cancer cells through the differential regulation of expression of the annexins, which are important calcium regulators. We have shown that p53 mediates pro-survival signalling in breast cancer cells and does not induce the canonical annexin A5 apoptotic pathway as previously thought. Although we, and others, have shown that reactivation of the canonical p14ARF-p53 pathway does not induce apoptosis in our studies, this does not necessarily relate to resistance to either chemotherapy or endocrine therapies. In our retrospective studies using a freely available breast cancer database, induction of p53 and overexpression of annexins associated with pro-survival functions is not associated with resistance to endocrine therapy. However, p53 induced overexpression of annexins, with consequent cellular phenotypic alterations appears to influence treatment outcomes in breast cancer. Importantly, prognosis/treatment outcome prediction is modified by whether one considers single genes individually or combines the gene expression profiles of various genes. Combining expression data of many genes is therefore the way forward to getting best /most accurate prognostic/treatment outcome information.

## Supporting Information

S1 Table Extract of annexin and S100A proteins from SILAC LC-MS/MS analysis. (XLSX)

S2 Table The gene description, function and process of the p53-p21-annexin/S100A interactome. (XLSX)
